# RETRACTION: Incidence Rate and Risk Factors of Surgical Wound Infection in General Surgery Patients: A Cross‐Sectional Study

**DOI:** 10.1111/iwj.70238

**Published:** 2025-02-25

## Abstract

**RETRACTION:** AshoobiM. T. , AsgaryM. R. , SarafiM. , FathalipourN. , PiroozA. , JafaryparvarZ. , RafieiE. , FarzinM. , SamidoustP. , and DelshadM. S. E. , “Incidence Rate and Risk Factors of Surgical Wound Infection in General Surgery Patients: A Cross‐Sectional Study,” International Wound Journal 20, no. 7 (2023): 2640–2648, 10.1111/iwj.14137.36896793 PMC10410328

The above article, published online on 10 March 2023, in Wiley Online Library (http://onlinelibrary.wiley.com/), has been retracted by agreement between the journal Editor in Chief, Professor Keith Harding; and John Wiley & Sons Ltd. A third party reported to the journal that they had found evidence of excessive self‐citations in the reference list of this article. The publisher did not confirm the evidence of excessive self‐citations. Upon further investigation, the publisher also concluded that this article was accepted solely on the basis of a compromised peer review process. In view of the clear evidence of compromised peer review, the parties agreed that the paper must be retracted. The authors did not respond to our notice regarding the retraction.

**RETRACTION:** M. T. Ashoobi , M. R. Asgary , M. Sarafi , N. Fathalipour , A. Pirooz , Z. Jafaryparvar , E. Rafiei , M. Farzin , P. Samidoust , and M. S. E. Delshad , “Incidence Rate and Risk Factors of Surgical Wound Infection in General Surgery Patients: A Cross‐Sectional Study,” International Wound Journal 20, no. 7 (2023): 2640–2648, 10.1111/iwj.14137.36896793 PMC10410328

The above article, published online on 10 March 2023, in Wiley Online Library (http://onlinelibrary.wiley.com/), has been retracted by agreement between the journal Editor in Chief, Professor Keith Harding; and John Wiley & Sons Ltd. A third party reported to the journal that they had found evidence of excessive self‐citations in the reference list of this article. The publisher did not confirm the evidence of excessive self‐citations. Upon further investigation, the publisher also concluded that this article was accepted solely on the basis of a compromised peer review process. In view of the clear evidence of compromised peer review, the parties agreed that the paper must be retracted. The authors did not respond to our notice regarding the retraction.

